# Pre- and Postnatal Exposure to Low Dose Glufosinate Ammonium Induces Autism-Like Phenotypes in Mice

**DOI:** 10.3389/fnbeh.2014.00390

**Published:** 2014-11-20

**Authors:** Anthony Laugeray, Ameziane Herzine, Olivier Perche, Betty Hébert, Marine Aguillon-Naury, Olivier Richard, Arnaud Menuet, Séverine Mazaud-Guittot, Laurianne Lesné, Sylvain Briault, Bernard Jegou, Jacques Pichon, Céline Montécot-Dubourg, Stéphane Mortaud

**Affiliations:** ^1^Immunologie et Neurogénétique Expérimentales et Moléculaires – UMR7355 CNRS – 3b, Orléans, France; ^2^Département de génétique, Centre Hospitalier Régional, Orléans, France; ^3^Université d’Orléans, Orléans, France; ^4^IRSET INSERM U 1085, Université de Rennes I, Rennes, France

**Keywords:** glufosinate ammonium, autistic spectrum disorders, pre- and postnatal exposure, neurodevelopment, mice

## Abstract

Glufosinate ammonium (GLA) is one of the most widely used herbicides in agriculture. As is the case for most pesticides, potential adverse effects of GLA have not been studied from the perspective of developmental neurotoxicity. Early pesticides exposure may weaken the basic structure of the developing brain and cause permanent changes leading to a wide range of lifelong effects on health and/or behavior. Here, we addressed the developmental impact of GLA by exposing female mice to low dose GLA during both pre- and postnatal periods and analyzed potential developmental and behavioral changes of the offspring during infancy and adulthood. A neurobehavioral test battery revealed significant effects of GLA maternal exposure on early reflex development, pup communication, affiliative behaviors, and preference for social olfactory cues, but emotional reactivity and emotional memory remained unaltered. These behavioral alterations showed a striking resemblance to changes seen in animal models of Autistic Spectrum Disorders. At the brain level, GLA maternal exposure caused some increase in relative brain weight of the offspring. In addition, reduced expression of *Pten* and *Peg3* – two genes implicated in autism-like deficits – was observed in the brain of GLA-exposed pups at postnatal day 15. Our work thus provides new data on the link between pre- and postnatal exposure to the herbicide GLA and the onset of autism-like symptoms later in life. It also raises fundamental concerns about the ability of current safety testing to assess risks of pesticide exposure during critical developmental periods.

## Introduction

Harmful effects of exposure to pesticides during critical developmental stages are becoming increasingly evident, and consequently, may significantly contribute to the rise in chronic diseases later in life such as cancer, infertility, diabetes, cardiovascular diseases, and obesity, but also in psychiatric disorders including schizophrenia, mood disorders, and autism spectrum disorders (Barouki et al., 2012). There is a growing interest in the range of alterations, which pre- and postnatal exposure to pesticides may produce on behavioral, emotional, and cognitive skills (Julvez and Grandjean, 2009). Previous studies reported that prenatal urinary measures of pesticides exposures were associated with increased abnormal reflexes in human neonates living in an agricultural community (Young et al., 2005). In line with this, prenatal exposure to some pesticides was associated with both psychomotor and mental developmental delays in young children (Rauh et al., 2006). Further, prenatal pesticide exposure has been associated with Autistic Spectrum Disorders (ASD) (Eskenazi et al., 2007).

Organophosphorus pesticides (i.e., compounds containing carbon–phosphorus bonds) are the most widely used pesticides in the world and comprise nearly 40 different chemical substances registered by the US Environmental Protection Agency (EPA) (http://www.epa.gov). According to the latest US EPA survey, glyphosate, a well-known compound in the organophosphorus family, was the most used herbicide in 2007 (EPA, 2011). As glyphosate-resistant weeds have begun to develop around the world (Cerdeira and Duke, 2006), herbicides containing glufosinate ammonium (GLA, another organophosphorus compound) will probably soon become the best alternative for treating glyphosate-resistant crops, and will therefore be used massively for years. To cope with this, many crop varieties have been genetically engineered to be GLA-tolerant (Ay et al., 2012). Accurate assessments of the adverse effects of GLA must therefore be conducted, in particular during the highly sensitive early stages of the life cycle.

Glufosinate ammonium is the ammonium salt of phosphinothricin [D,L-homoalanin-4-(methyl) phosphinate], a structural analog of glutamate. GLA acts as a competitive and irreversible inhibitor of glutamine synthetase, a vital enzyme in plants, needed to fix nitrogen to organic molecules of ammonium ions. However, glutamine synthetase is also present in the mammalian central nervous system (CNS). In astroglial cells, glutamine synthetase plays an essential role in the homeostasis of glutamate, a major neurotransmitter in the CNS (Bak et al., 2006). In acute conditions, GLA has neurotoxic effects, as evidenced by neurological symptoms (seizures and memory loss) displayed by humans attempting suicide by ingesting large quantities of GLA-containing herbicide (Park et al., 2006). In animal models, GLA has been shown to induce seizures in mice (Lapouble et al., 2002). Additionally, it has been shown that GLA-induced embryo lethality, major morphological head defects (Watanabe and Iwase, 1996), and neurepithelium apoptosis (Watanabe, 1997) in developing mouse embryos in culture. Direct exposure of the rat brain to acute doses of GLA causes alterations to glutamate neurotransmission (Nakaki et al., 2000). We recently showed that chronic administration of a moderate dose of GLA-induced memory impairments, brain structural modifications, astrogliosis, and disturbances of the glutamate homeostasis in adult mice (Calas et al., 2008; Meme et al., 2009).

While the neurotoxic effects of acute doses of GLA in adult humans are well documented, little is known about the chronic effects of low doses of GLA during neurodevelopmental stages. It is essential to characterize the harmful long-term effects, which may interfere with brain development and lead to permanent abnormalities. Here, we developed an experimental murine model of chronic pre- and postnatal exposure to low dose GLA and examined the down-stream effects, at the behavioral and molecular levels in pups and adult mice. Ontogenetic endpoints typical of infancy such as ultrasonic calls and early reflex development were assessed early after birth. We further investigated the long-term effects on a number of behavioral dimensions during adulthood. We explored gene expression in the brain to establish whether such changes could be related to GLA-induced behavioral abnormalities. We chose to administer GLA intranasally as very few studies are exploring the effects of aerosol exposure to pesticides despite accumulating evidence that air is particularly relevant to agricultural communities that are highly exposed to pesticides by air contamination (Fenske et al., 2002a,b). Urban people may also be concerned by such a pollution as non-dietary exposure to pesticides occurs mostly in the home (Simcox et al., 1995), as evidenced by studies on urban settings highlighting the widespread use of pesticides indoors (Whyatt et al., 2002; Berkowitz et al., 2003).

## Materials and Methods

### Animals and treatments

Seven-week-old female C57Bl6 mice were purchased from Janvier (Le Genest St Isle, France). All mice were bred and maintained on a 12-h light/dark cycle (lights on from 7:00 a.m. to 7:00 p.m.) with food and water *ad libitum* in a temperature controlled (21 ± 1°) room in the animal resource facility (UPS44, CNRS Orléans – France). After an acclimation period of 2 weeks, two female mice were paired with one male C57Bl6 mouse also from Janvier (Le Genest St Isle, France), in standard laboratory cages (42 cm × 28 cm × 18 cm) to mate. Pregnant mice were then isolated and divided into three experimental groups treated intranasally with GLA (1 or 0.2 mg/kg; PESTANAL^®^, analytical standard from Sigma–Aldrich) (GLA1 and GLA0.2) and the control group (CTL) with saline solution (NaCl 0.9%). Intranasal exposure was performed by administration of 10 μL of solution for a 30 g mouse deposited at the entrance to the nostrils. Dams were treated three times a week, from gestational day (GD) 10 to postnatal day 15 (PND 15). Control animals received a comparable dose of 0.9% saline. Offspring were weaned at 21 days of age and maintained in same-sex, litter-mate housed cages with *ad libitum* access to food and water. All aspects of animal care and experimentation were in accordance with the European Parliament and Council Directive (2010/63/EU). The Ethics Committee approved all animal care and use for this study (Approval C45-234-6).

### General procedure

**Figure 1:**
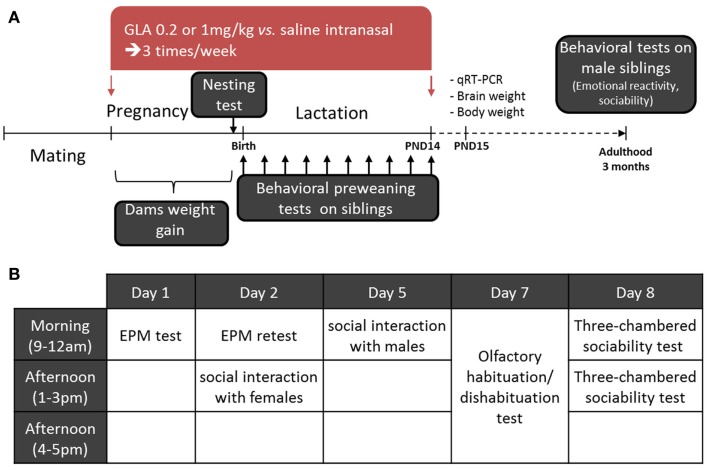
**Experimental design of the study**. **(A)** On arrival, female mice were kept undisturbed in the laboratory for 2 weeks before beginning the experiment. They were then paired with male mice for 5–6 days to mate. Pregnant mice were treated intranasally with either GLA (0.2 or 1 mg/kg) or saline solution. Dams were treated three times a week until postnatal day 14 (PND 14). During pregnancy, female mice were weighed and their ability to build a nest tested 2–3 days before delivery. Preweaning tests were performed on all siblings from birth to PND 14. Males only were then left undisturbed until adulthood (3 months). A second cohort was left reaching PND 15 and was then euthanized to perform whole brain qRT-PCR and body/brain weight analyses. **(B)** At adulthood, the male mice were tested as follows: on day 1, the elevated plus maze (EPM); day 2, EPM retest and social interaction with females; day 5, social interaction with males; day 7, the olfactory habituation/dishabituation test; and day 8, the three-chambered sociability test.

The general procedure is shown in Figure 1A. Mating was performed within 5–6 days. At the end of this period, males were removed and dams were weighed every 2 days. Females having gained 2 g were considered pregnant and were then isolated in standard cages including nesting material (pressed cotton). Intranasal treatment began the same day and lasted until PND 15. Each female received 10 treatments (4 during pregnancy and 6 during lactation) three times a week (on Monday, Wednesday, and Friday; between 9:00 and 12:00 a.m.). By using such a protocol, we could notice that about 10 ± 1 day separated the first exposure from delivery. Thus, we could estimate the exposure to begin between GD 9–11 for all the litters. In order to verify that intranasal exposure to GLA did not induce olfactory deficits in dams (which could be responsible for abnormal maternal behavior), we exposed another group of non-pregnant C57Bl6 females to a similar protocol of administration (10 intranasal treatments with CTL, GLA0.2, and GLA1 solutions) and tested their olfactory abilities in the habituation/dishabituation olfactory test (see below for detailed procedure).

As previously stated, females were weighed every 2 days during pregnancy (before each treatment) in order to evaluate GLA toxicity during gestation. Two to three days before delivery, we assessed females’ nesting abilities to check whether GLA-induced behavioral deficits. At birth, the mean number of pups by litter was determined. One day after birth, all pups irrespective of their sex, were weighed and then tested in a series of preweaning tests to check the effects of pre- and postnatal exposure to GLA on postnatal development. As GLA is not known to have sex-specific effects, we chose to not differentiate male from female siblings for early postnatal analyses. Indeed, our goal was rather to detect gross developmental alterations. The set of analyses performed is recapitulated in Table 1. On a first cohort, we investigated pups communication by monitoring of ultrasonic vocalizations (USVs) from PND 1 to 5. Immediately after the 3-min period of USV collecting, we assessed early sensory and motor development by monitoring seven parameters in all pups: the righting reflex (from PND 1 to 10), walking and 45° negative geotaxia (from PND 5 to 10), vertical climbing and bar grasping (from PND 9 to 14), and the day of eyelid opening and acquisition of the acoustic startle reflex (from PND 11 to 14). Duration of this testing period was no more than 5 min by pup. Data from each pup of all litters (*n* = 8 litters for CTL and *n* = 6 litters for GLA0.2 and GLA1) was collected and then averaged so as to the litter was used as the unit for statistical analyses. At weaning time (PND 21), only male offspring were reared by litter and left undisturbed until the age of 3 months to conduct adult behavioral analyses. For adult testing, groups’ composition was as follow: *n* = 28 (from eight litters) for the CTL group, *n* = 19 (from six litters) for the GLA0.2 group, and *n* = 11 (from four litters). We chose to focus on male offspring in order to avoid hormonal fluctuations occurring during the estrous cycle, which could affect animal behavior and thus complicate data interpretation. Male offspring were tested in the elevated plus maze (EPM) to assess emotional reactivity, in the 24-h EPM retest to assess emotional memory, in the three-chambered sociability and social interaction test (vs. female and vs. male) to assess sociability, and in the olfactory habituation/dishabituation test to assess their preference for social olfactory cues. All the tests were performed as shown in Figure 1B. Another set of pups pre- and postnatally exposed to CTL, GLA0.2, or GLA1 solutions were allowed to reach PND 15 in order to perform physical analyses (brain and body weight) and gene expression analyses by qRT-PCR from whole brain tissues. For these analyses, only male pups were used.

**Table 1:** **Analyses performed on control and glufosinate exposed offspring**.

	PND 1	PND 2	PND 3	PND 4	PND 5	PND 6	PND 7	PND 8	PND 9	PND 10	PND 11	PND 12	PND 13	PND 14	PND 15	Adulthood
**Behavioral analyses (Cohort 1)**
Ultrasonic vocalizations	*	*	*	*	*											
Righting reflex	*	*	*	*	*	*	*	*	*	*						
Walking					*	*	*	*	*	*						
45° Negative geotaxia					*	*	*	*	*	*						
Vertical climbing									*	*	*	*	*	*		
Bar grasping									*	*	*	*	*	*		
Eyelid opening											*	*	*	*		
Acoustic startle reflex											*	*	*	*		
Elevated plus maze test															*	*
Three-chambered sociability test															*	*
Interaction test vs. female															*	*
Interaction test vs. male															*	*
Habituation/dishabituation olfactory test															*	*
**Molecular analyses (Cohort 2)**
Whole brain qRT-PCR															*	
**Physical analyses (Cohort 1, 2)**
Body weight	*														*	
Brain weight															*	

*On a first cohort, pups communication was investigated by monitoring of ultrasonic vocalizations (USVs) from PND 1 to 5. Immediately after, early sensory and motor development was assessed by monitoring seven parameters in all pups. At weaning time (PND 21), male offspring were tested in the elevated plus maze (EPM) to assess emotional reactivity, in the 24-h EPM retest to assess emotional memory, in the three-chambered sociability and social interaction test (vs. female and vs. male) to assess sociability, and in the olfactory habituation/dishabituation test to assess their preference for social olfactory cues. Another set of pups pre- and postnatally exposed to CTL, GLA0.2, or GLA1 solutions were allowed to reach PND 15 in order to perform physical analyses (brain and body weight) and gene expression analyses by qRT-PCR from whole brain tissues*.

### Olfactory abilities of female mice intranasally exposed to CTL/GLA solutions

Non-pregnant C57Bl6 females were exposed three times a week to intranasal treatments with CTL (*n* = 8), GLA0.2 (*n* = 8), and GLA1 solutions (*n* = 8) during a time period corresponding to the treatment period of dams in the pre- and postnatal exposure protocol (10 treatments). Their olfactory abilities were then tested in the habituation/dishabituation olfactory test (see experimental procedure described below) 24 h after the last treatment.

### Neurobehavioral assessment: Preweaning tests

#### Ultrasound vocalizations

Vocalizations were recorded (from PND 1 to 5; each animal was tested on each of these days) during a 3-min period using the following experimental setup: a custom designed recording chamber made of transparent Plexiglas, four ultrasound detectors (UltraVox 4-channel system; Noldus Information Technology), and data acquisition software (UltraVox 2.0; Noldus Information Technology), which automatically monitored the occurrence of vocalizations within user-defined frequencies (in our case: 20, 40, 60, and 80 KHz). The number of USVs was recorded on a personal computer for offline analysis and storage. The computerized recording system was set to eliminate non-relevant sounds (background noise) and to ignore ultrasounds outside the defined frequency range. Ultrasonic calls were recorded for 3-min periods, between 9:00 and 12:00 a.m., in an experimental room maintained at approximately 21°C. A single pup was removed from the litter and placed in a square container (5 cm × 5 cm; height, 2 cm) at the center of a sound-attenuating chamber, and USVs were assessed. All the pups were individually tested in turn. The container had been saturated with maternal odor to avoid environmental stress and had been cleaned between runs. After the 3-min recording session, each pup went through all the other preweaning tests. For each pup, we measured the cumulative number of calls emitted during the 5 days (i.e., total number of calls from PND 1 to 5). The litter was used as the unit for statistical analyses.

#### Postnatal sensory and motor development

The investigation of early postnatal sensory and motor development monitored seven parameters:

– *Negative geotaxia*: The pup turned upwards when placed on a 45° angle slope with its head pointing down the incline (PND 5–10). We recorded the age when pups acquired the reflex.
– *Adult walking pattern*: During the first days of the pup’s life, the steps produce a pivoting walk, later developing into linear displacement. We recorded the age when linear displacement replaced pivoting (PND 5–10).
– *Righting reflex*: When a pup is placed on its back on a flat, hard surface, it has to right itself on all four paws, with two successes in three trials getting a score of 1. A failure is recorded when the pup remains on its back for more than 10 s, and a score of 0 is given. The age when pups got two successes in three trials was recorded (from PND 1 to 10).
– *Vertical climbing*: The pup is held against a vertical metallic grid (mesh: 6 mm wide). Climbing was scored when the pup passed out five stitches (PND 9–14). The day of success was recorded.
– *Bar Holding*: The forepaws are placed on a round wooden bar (7 mm diameter). The ability to hang for 10 s using the forepaws was scored (PND 9–14). The day of success was collected.
– *Eyelid opening*: Defined as any visible break in the membrane covering the eye. We examined pups from PND 11 to 14 and determined the day of eyelid opening (pups with at least one eyelid opened).
– *Acoustic startle reflex*: From PND 11 to 14, a small hand-held clicker generated a loud noise and the jerk behavior immediately following was scored. The day of reflex acquisition was noted.

For all these tests, each pup was tested and their scores averaged per litter so as to the litter was used as the unit for statistical analyses.

### Adult behavior

#### Three-chambered sociability test

Mice were run individually in a three-chambered arena made of clear polycarbonate. Retractable doorways built into the two dividing walls controlled access to the side chambers. Each of the two outside chambers had an inverted empty wire cup, one side housing a male CBA/J stimulus mouse age-matched to the test mouse, and the other side with a plastic object (plastic mouse). The test session began with a 5-min habituation session for the entire apparatus with the mouse free to explore the entire arena. The subject was then briefly confined to the center chamber while the plastic object was placed in the cup on one side and an adult male CBA/J mouse was placed in the cup on the other side. The object/novel mouse sides were alternated left and right between subjects. Once the stimuli were in position, the two side doors were simultaneously raised and the subject could access all three chambers for 5 min. Automatic monitoring recorded and scored the time spent in contact with each wire cup using the EthoVision video-tracking system (Noldus, The Netherlands). The apparatus was cleaned between subjects using a 70% ethanol/water solution.

#### Social interaction

Social interaction was tested in a novel cage (24 cm × 11 cm × 12 cm) with clean sawdust. Each mouse was paired with an unfamiliar NMRI female on day 1 and with an unfamiliar CBA/J male mouse on day 2. Opponent mice were age-matched to the test mouse. The test mouse was isolated for 30 min before the unfamiliar conspecific was placed in the cage. Social interaction between the two was recorded using a digital camera mounted above the cage at ceiling level. The paired mice had never interacted before. The videos were analyzed by an independent observer blind to the study protocol. Social investigation was scored according to the following behavioral parameters:

– head, body, and anogenital sniffing: sniffing of the conspecific by the test mouse
– walkover: the test mouse places its forepaws on the head or back of the conspecific
– following: the test mouse follows the conspecific directly behind it.
– mounting
– allogrooming: the test mouse grooms the conspecific
– aggression.

The duration of the social interaction was calculated as the total time spent by the test mouse carrying out all or any of the above-mentioned behaviors.

#### Habituation/dishabituation olfactory test

The test was an adapted version of Yang and Crawley (2009). Each subject mouse was tested in a clean arena with a thin layer of fresh bedding. Cotton swab material was used as the medium for each odor stimulus and was inserted in a small plastic tube (Eppendorf; 1 mL) cut open at the bottom (see Video 1 in Supplementary Material). To reduce novelty-induced exploratory activities, a 30 min period was set aside before testing for each subject to become habituated to the testing cage, which contained a clean dry cotton swab.

Testing consisted in a sequence of 15 × 2-min exposures to odors presented in the following order: 3 plain tap water, 3 almond, 3 banana, 3 social odor 1, and 3 urine/social odor 2. The almond and banana odors were made with food flavoring (Selectarôme, Cannes La Bocca, France). All non-social odors were made by dipping the cotton into the solution for 1 s. Social odors were made by mixing urine from a number of C57Bl6 adult male mice. Each subject was exposed to two novel social odors made from two different sources. Time spent sniffing the tube was measured using the EthoVision video-tracking system (Noldus, The Netherlands). Sniffing was scored when the distance between the nose and the tube was 1.5 cm or less. Each inter-session interval was approximately 1 min.

#### Elevated plus maze

The EPM was used as it is known to detect anxiety-like behavior in rodents (Pellow et al., 1985; Yee et al., 2007). The apparatus had a central hub (5 cm × 5 cm), and two pairs of diametrically opposed arms, one pair open with no sides (27 cm × 5 cm) and the other pair fully enclosed (27 cm × 5 cm × 15 cm). The maze was made of black Plexiglas, elevated to a height of 60 cm and the open arms were lit by bulbs providing 35 lux at the far end of each. The 5-min test began by placing an individual mouse at the end of an enclosed arm. A video camera mounted above the maze recorded the trials for later analysis. The apparatus was cleaned between runs with a 70% ethyl alcohol solution. The number of entries into and the time spent on the anxiogenic open arms were used as conventional spatiotemporal measures of avoidance. The time spent in the central hub area and the total distance traveled in the apparatus were also measured. All the analyses were performed using the EthoVision^®^ video-tracking system (Noldus Inc., The Netherlands). Twenty-four hours after the first trial, each animal was retested in the same apparatus. It is known that the EPM retest effect can show a biologically adaptive form of learning/memory where the experience of a novel environment will swiftly lead to less time being spent in the unsafe open arms (Bertoglio et al., 2005).

### Quantitative RT-PCR

Paternally expressed gene 3 and Pten Gene expression data were obtained from male offspring only (PND 15). Animals were euthanized by CO_2_ inhalation and their brain collected for analyses. Total RNA was isolated from whole brain tissue homogenate using Trizol reagent (Ambion, Life technologies, Villebon-sur-Yvette, France) and was quantified by spectroscopy. One microgram of total RNA was reverse transcribed with 100 units of Superscript III reverse transcriptase (Invitrogen, Life Technologies, Villebon-sur-Yvette, France). Real-time PCR reactions were performed in the Mx3005P Agilent (Applied, Life technologies, Villebon-sur-Yvette, France) with fivefold dilution of cDNA, 200 nM of each Taqman primer using Gene Expression Master Mix (Life Technologies, Villebon-sur-Yvette, France). Data were analyzed using the DDCt method and normalized against control samples with mice Gapdh and 18S (Perche et al., 2013). Amplification efficiencies of the target gene and the reference gene are identical. Each measurement was performed three times. The values for the two treated group (GLA 1 and 0.2 mg/kg) were normalized to controls.

### Statistical analysis

Litter was used as the unit of analysis of USVs, all early sensorimotor endpoints and body weight data. Such data were analyzed using non-parametric procedures specially adapted for statistical analysis of small samples (*n* < 30). These statistical procedures have two main advantages over the parametric approach: (1) they do not depend on population assumptions and therefore do not run the risk of not meeting the prerequisite criteria (Siegel and Castellan, 1988), and they have better relative power-efficiency for small samples (Bridge and Sawilowsky, 1999). Independent two-group comparisons were analyzed using the Mann–Whitney *U* test. When considering more than two groups, a global analysis was done first using the Kruskal–Wallis “ANOVA-on-ranks” procedure, then, when necessary (*p* < 0.05), by Bonferroni *post hoc* analyses (Siegel and Castellan, 1988) including the correction for multiple comparisons. Percentage measurements were analyzed for differences using Chi-squared statistics. qRT-PCR and adult behavioral data were also analyzed using above-mentioned non-parametric procedures.

## Results

### GLA did not induce toxicity in dams

**Figure 2:**
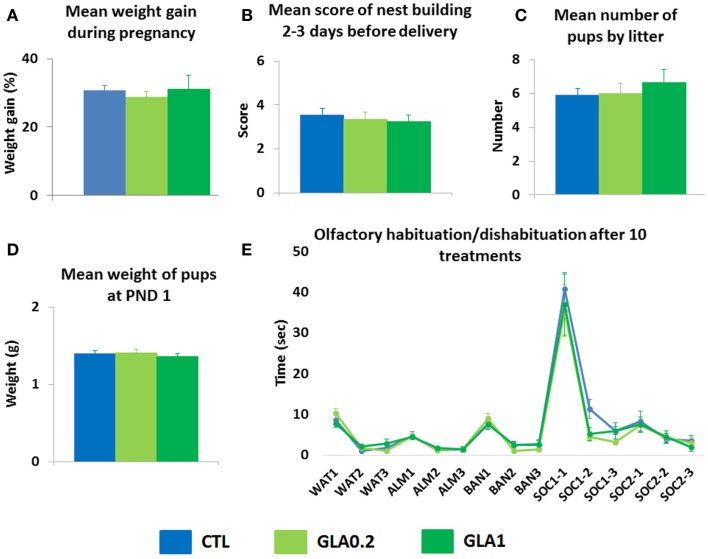
**Pre- and postnatal glufosinate ammonium exposure did not alter physiology/behavior in exposed dams**. **(A)** GLA did not modify the percentage of weight gain in pregnant mice. **(B)** GLA did not impair dams’ ability to build a nest 2–3 days before delivery data are the mean ± SEM (*n* = 6–8 dams/group). In addition, GLA did not induce toxic effects on pups during pregnancy as **(C)** the mean number of pups by litter was not different between groups and **(D)** the mean weight of pups at PND 1 was not affected by GLA exposure. Data are the mean ± SEM (*n* = 6–8 litters/group). **(E)** Intranasal instillation of GLA solutions three times per week did not induce any olfactory deficits in non-pregnant females exposed to the same number of treatments than pregnant ones (10 treatments). In this task, cotton swabs were used to deliver olfactory stimuli: either non-social (water, almond, and banana) or social (two different odors/cues containing a mixture of urine from a number of male mice). The odor/swab was inserted into a small plastic tube cut open at the bottom. Control mice displayed normal olfactory function with classical habituation/dishabituation to both non-social and social odors. They also displayed a clear preference for social olfactory cues. GLA1 mice displayed. Data are the mean ± SEM (*n* = 8/group).

In order to verify that exposure to GLA did not induce adverse effects in dams, we monitored maternal physiological and behavioral parameters both during pregnancy and postnatally. We observed that neither maternal weight gain nor nest building abilities few days before delivery were altered by GLA exposure during pregnancy (Figures 2A,B). In addition, neither the mean number of pups by litter nor their body weight at PND 1 were changed in GLA-exposed offspring compared to controls (Figures 2C,D). We also found that intranasal exposure to GLA did not result in abnormal olfactory skills as non-pregnant C57Bl6 mice exposed to a similar protocol of administration did not display any deficits in the olfactory habituation/dishabituation test (Figure 2E).

### GLA-induced developmental deficits in pre- and postnatally exposed offspring

Investigating the possibility of GLA affecting development, we first determined whether pre- and postnatal exposure to GLA caused abnormal sensorimotor skills in offspring. We focused on three parameters: communication skills, sensorimotor development, and gross developmental features (body/brain weight).

#### Communication skills

**Figure 3:**
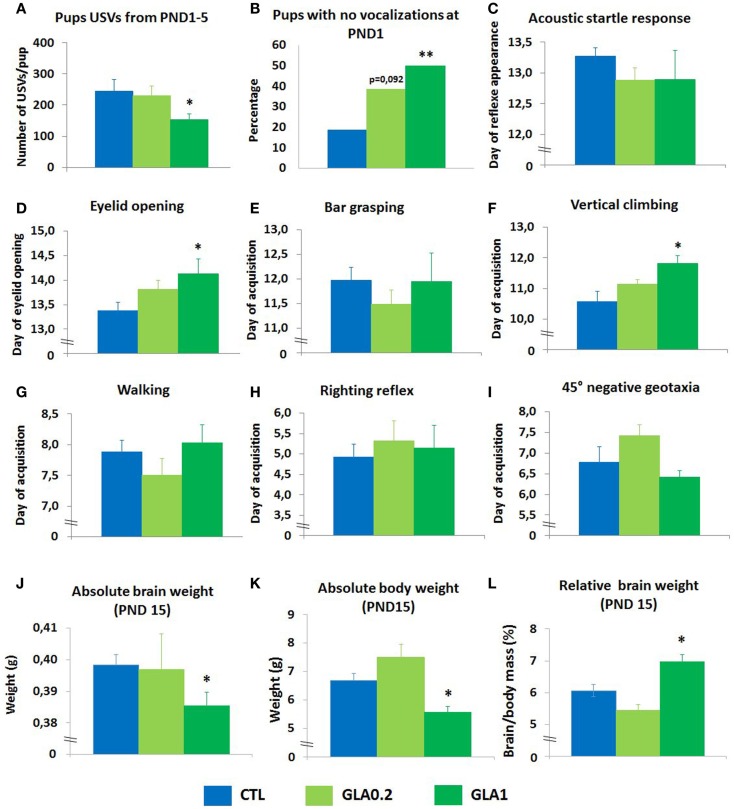
**Pre- and postnatal glufosinate ammonium exposure disturbed neurodevelopmental endpoints**. **(A)** Mean total number of vocalizations emitted by pups when separated from their mother from postnatal day 1 to postnatal day 5. Only GLA 1 mg/kg caused a drop in the number of ultrasonic vocalizations emitted by pups when separated from their mothers. Data are the mean ± SEM (*n* = 6–8 litters/group). **(B)** At postnatal day 1, GLA-exposed mice display dose-dependent communication deficits. Data are presented as the percentage of pups emitting no vocalizations when separated from their mothers. Chi-square analysis was used. **(C)** Acoustic startle reflex data are presented as the average day when pups displayed startle response. Data are the mean ± SEM, *n* = 6–8 litters/group. **(D)** Eyelid opening data are presented as the average day when pups had at least one eye open. Data are the mean ± SEM, *n* = 6–8 litters/group. **(E)** Bar grasping reflex, **(F)** vertical climbing, **(G)** walking reflex, **(H)** righting reflex, and **(I)** 45° negative geotaxia data are presented as the average day when pups were successful in the task. Data are the mean ± SEM, *n* = 6–8 litters/group. Data indicate that only GLA1-exposed pups display deficits in the acquisition of certain reflexes. At postnatal day 15, neither absolute brain weight **(J)** nor the absolute body weight **(K)** were affected by the treatment while relative body weight **(L)** was significantly increased in GLA1 offspring and slightly decreased in GLA0.2 offspring compared to controls. *p < 0.05; **p < 0.01, when compared to control mice (CTL). Significant *p* values are Bonferroni corrected.

Ultrasonic vocalizations by neonatal mice have been studied both as a manifestation of early communicative behavior of the pup–mother dyad and as a sign of an aversive affective state (Wohr and Schwarting, 2008). We analyzed the pups’ communication skills in response to a 3-min period of separation from the mother, measuring the total number of ultrasound vocalizations emitted from postnatal day (PND) 1 to 5. At the higher dose (1 mg/kg; GLA1), GLA significantly decreased the number of calls emitted (*p* = 0.045) (Figure 3A). No such alterations were observed at the lower dose (0.2 mg/kg; GLA0.2). Total duration of calls was also reduced in GLA1 offspring only (Table S1 in Supplementary Material). Moreover, the proportion of GLA-exposed pups emitting no vocalizations at PND 1 was greater than for the control pups (*p* = 0.006 for GLA1 and *p* = 0.092 for GLA0.2) (Figure 3B).

#### Sensorimotor development

We analyzed the effects of GLA exposure on the timing of several patterns of sensorimotor development. Acquisition of the acoustic startle reflex was not altered by GLA exposure (Figure 3C). Eyelid opening was delayed in the GLA1 group whereas no effect was noted in GLA0.2 treated offspring (Figure 3D). No effects of GLA were observed on bar grasping (Figure 3E), walking (Figure 3G), righting reflex (Figure 3H), and negative geotaxia (Figure 3I). However, a delayed acquisition of the vertical climbing reflex was observed at the highest dose (*p* = 0.034) (Figure 3F). No effect was observed for the GLA0.2 group.

#### Brain anatomy

We studied the question of whether gross developmental features might be affected by GLA, doing so by measuring the brain and body weight of the pups at PND15. Observations showed that, at PND15, both the absolute brain weight and the absolute body weight were significantly reduced in GLA1 treated offspring compared to controls (Figures 3J,K). No effect was seen in GLA02 treated offspring. Calculation of the relative brain weight indicated that pre- and postnatal GLA exposure significantly increased the relative brain weight in mice given the higher dose (*p* = 0.046) while mice given the lower dose displayed slight decrease of the relative brain weight (*p* = 0.09) (Figure 3L).

Thus, the highest dose of GLA (1 mg/kg) induced developmental deficits in pre- and postnatally exposed offspring, affecting communication skills and sensorimotor development in early postnatal life, and changing the relative weight of the brain.

### Effects of GLA on offspring lasting into adulthood

To investigate whether these effects on developmental milestones might translate into long-lasting consequences on adult behavior, we analyzed the behavioral repertoire of 3 months old offspring by assaying social parameters namely the three-chambered sociability test, the social interaction test and the olfactory habituation/dishabituation test, and also emotional skills in the elevated plus maze test.

#### Social skills

**Figure 4:**
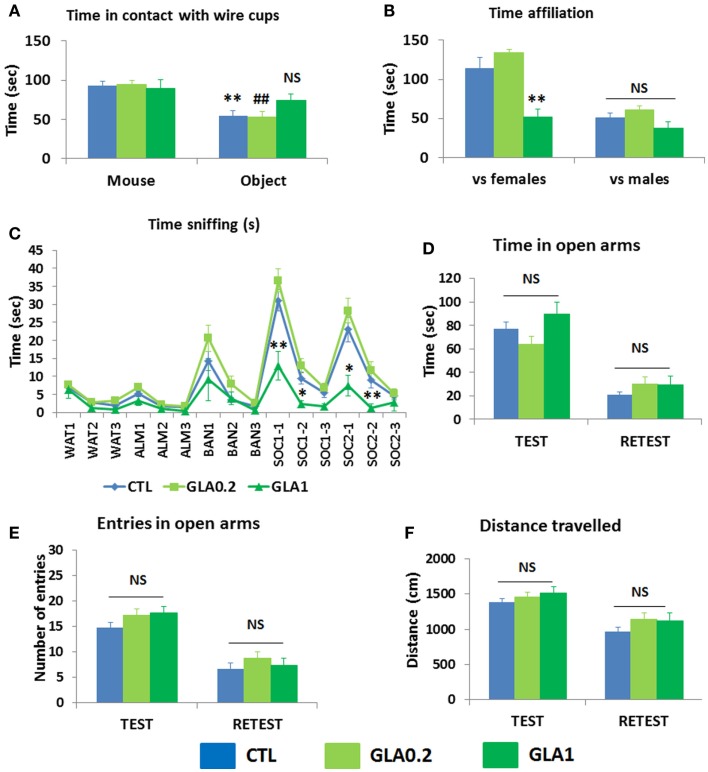
**Pre- and postnatal glufosinate ammonium exposure resulted in behavioral alterations in the offspring during adulthood**. **(A)** GLA1 offspring displayed no preference for the chamber containing the social partner, unlike the controls, in the three-chambered sociability test. No effect of GLA0.2 was observed. **p < 0.01 compared to time spent in contact with wire cup containing the mouse in the CTL group; ^##^*p* < 0.01 compared to time spent in contact with wire cup containing the mouse in the GLA0.2 group. **(B)** In the social interaction test, GLA1 mice confronted with females interacted less, but no difference was observed when they were confronted with males. **(C)** In the habituation/dishabituation olfactory test, cotton swabs were used to deliver olfactory stimuli: either non-social (water, almond, and banana) or social (two different odors/cues containing a mixture of urine from a number of male mice). The odor/swab was inserted into a small plastic tube cut open at the bottom. Control mice displayed normal olfactory function with classical habituation/dishabituation to both non-social and social odors. They also displayed a clear preference for social olfactory cues. GLA1 mice displayed significantly less interest in social cues, but this was not the case for GLA0.2 mice. **(D–F)** GLA did not alter offspring’s behavior in the elevated plus maze. All data are presented as means ± SEM, *n* = 11–27 mice/group; *p < 0.05; **p < 0.01, compared to control mice.

The three-chambered sociability test was used to investigate animals observing voluntary initiation of social interaction and their ability to discriminate social novelty. The animal was placed in a cage and left to explore and initiate social contact with a partner inside a wire container or an identical container holding a novel object. As expected, the control mice spent more time in interaction with the container with the mouse than with the container with the object (*p* = 0.0056; Figure 4A). GLA1 mice did not spend any more time with the container with the mouse than with the container with the object (Figure 4A). GLA0.2 treated mice, however, had the same response as the controls, displaying more time interacting with the container with the mouse than with the container with the object (*p* = 0.009; Figure 4A).

To further document social skills impairment, we paired GLA-exposed mice with both an unfamiliar female on day 1 and an unfamiliar male mouse on day 2, and further explored their social abilities in a more naturalistic way. Observations showed that GLA altered male–female interaction: GLA1 mice recorded less affiliation time with the female than the controls (*p* = 0.0091); male–male interaction was not affected (Figure 4B). No alterations were observed in GLA0.2 exposed offspring (Figure 4B).

Social interactions in rodents are based on very complex processes involving efficient detection, recognition, and processing of olfactory information (Vosshall, 2005). We thus conducted an olfactory habituation/dishabituation test known to assess the olfactory system of the mouse, the preference for social rather than non-social olfactory cues and learning/memory skills related to both social and non-social odors (Silverman et al., 2010). Our findings were than that the administration of GLA, both at 1 and 0.2 mg/kg, had no effect on olfactory discrimination skills: the mice were all able to detect new odors of all types, both non-social and social (Figure 4C). GLA did not disturb mice ability to habituate to both social and non-social odors encountered shortly before (Figure 4C). More importantly, compared to controls, GLA1 mice displayed significantly less interest in social olfactory cues, although performance on non-social odors was the same; this effect was not observed in GLA0.2 mice (Figure 4C).

#### Emotional skills

After exploring sociability in mice, we investigated emotional reactivity. Testing GLA-exposed mice and controls in the EPM, no robust differences were found as no effects of GLA were observed on the number of entries and time spent in the open arms (Figures 4D,E). Emotional memory was assessed by retesting mice 24 h later in the same apparatus. In the retest trial, mice normally avoid open arms and show less interest in exploring the entire apparatus (Rodgers et al., 2011). This is what we observed in CTL, GLA0.2, and GLA1 mice (Figures 4D,E). Moreover, distance traveled by GLA0.2 and GLA1 mice was similar to that traveled by CTL mice (Figure 4F), both during test and retest trials.

Thus, pre- and postnatal exposure to GLA was associated with behavioral changes in offspring that lasted during adulthood and that may be specific to social interaction.

### GLA-induced alterations are associated with altered expression of genes involved in autism-like phenotypes

**Figure 5:**
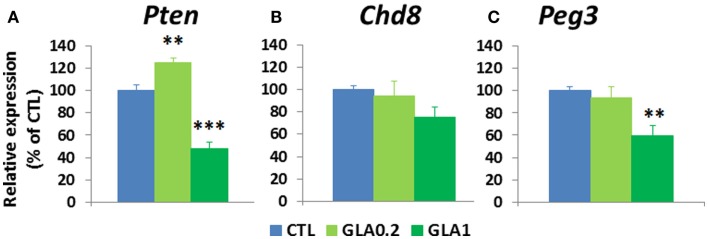
**Pre- and postnatal glufosinate ammonium exposure altered molecular parameters in the offspring brain at postnatal day 15**. **(A)** Whole brain quantitative RT-PCR analysis of *Pten* revealed that GLA0.2 increased while GLA1 reduced *Pten* transcripts in comparison to controls. **(B)** Whole brain expression of *Chd8* was not changed in GLA1 and GLA0.2 mice. **(C)** *Peg3* transcripts were reduced only in GLA1 mice. Each value is the mean ± SEM (*n* = 4–7 pups/group). **p < 0.01, ***p < 0.001. Significant *p* values are Bonferroni corrected.

It is known that a large number of genes are involved in brain weight changes and disturbed social behaviors (Delorme et al., 2013). This is the case for the *Pten* (phosphatase and tensin homolog) gene; *Pten* deregulation has been associated with changes affecting brain weight (Page et al., 2009) and social behavior (Page et al., 2009). Our initial working hypothesis was that the administration of GLA could affect *Pten* expression. Quantitative RT-PCR (qPCR) was conducted to measure whole brain mRNA levels of *Pten* at PND 15 in male controls vs. GLA-treated offspring. We found that pre- and postnatal exposure to GLA1 significantly reduced *Pten* expression, but the opposite effect was observed for the mice given the lower dose of GLA (Figure 5A). Other common gene mutations have been found to be related to anomalies of brain weight in certain cases of sporadic ASD in humans (Klein et al., 2013), e.g., for the Chromodomain Helicase DNA-binding-8 (*Chd8*) gene (O’roak et al., 2012). We therefore measured the level of *Chd8* expression in the whole brain of GLA-treated male offspring at PND 15 by qPCR. We found *Chd8* to be only slightly down-regulated in GLA1 mice compared to control mice but this effect was not statistically significant. No change was observed in GLA0.2 mice (Figure 5B).

Among genes involved in the regulation of social behavioral patterns, few are specifically related to male–female interactions. The Paternally Expressed Gene 3 (*Peg3*) has been shown to play a key role in the regulation of male–female affiliative behaviors (Swaney et al., 2008). We therefore studied the effect of pre- and postnatal administration of GLA to whole brain mRNA levels of *Peg3*, measured at PND 15 in male offspring. *Peg3* expression was significantly down-regulated in GLA1 mice, although no change was observed in GLA0.2 mice (Figure 5C).

Thus, pre- and postnatal exposure to GLA alters the developmental expression of genes related to ASD-like symptoms.

## Discussion

The major concern of this kind of study (pre- and postnatal exposure) is the putative toxic effects of GLA on maternal physiology and/or behavior. Indeed, we cannot totally rule out this possibility, in particular regarding long-term effects of pre- and postnatal exposure to GLA on social behaviors in the offspring. Indeed, it is now well-known that early social environment (maternal care and peer interactions) has a profound impact on offspring’s abilities in building normal social competencies (Branchi et al., 2013). However, we collected physical and behavioral data during pregnancy showing that mothers exposed to GLA were not different from their control counterparts as maternal weight gain and nest building capacities were not changed by the treatment. Consequently, behavioral changes observed in GLA-exposed offspring were not likely to result from any physiological and or behavioral deficits induced in mothers. Similarly, long-term effects of pre- and postnatal exposure to GLA could be the consequence of a direct effect of GLA on females’ olfactory abilities given that it was instilled intranasally. Indeed, in mice as in many other mammals, olfactory cues are extensively used in many aspects of maternal care to ensure the coordination of mother–infant interactions and consequently the normal development of the offspring. Therefore, disturbed perception of the smell of the offspring could result in impairments in maternal care and contribute to abnormal sociability during the adulthood of the offspring (Starr-Phillips and Beery, 2014). However, data collected in non-pregnant mice exposed to a similar protocol of treatment with GLA, showed that this procedure was not harmful and thus, was unable to be the cause of the developmental and/or long-term behavioral changes observed in GLA-exposed offspring.

Isolation-induced vocalizations are important communication signals eliciting maternal care behaviors. This type of behavior has received particular attention in respect of human language understanding. Indeed, there have been a number of study indicating specific pup and adult USV irregularities in mice with alterations in genes such as FOX P2 (Shu et al., 2005) and oxytocin gene OXT (Liu et al., 2008) (i.e., implicated in language and social behavior, respectively) as well as neuroligin-4 and neuroligin-3, which have been associated with ASD (Fischer and Hammerschmidt, 2011). According to some authors, this type of behavior might be reflective of the animal’s drive to communicate (Holtzman et al., 1996). In addition, pups USVs have also been involved in emotionality, an increase in vocalizations being observed in response to anxiogenic drugs, and a decrease in response to anxiolytics (Hodgson et al., 2008). Thus the reported reduction of USVs in GLA-exposed offspring may be evidence of disturbances in neuronal networks underlying emotional processes. However, the fact that no changes were observed in anxiety levels in adult offspring suggested that the neural circuitry underlying emotion-based behaviors was not affected by pre- and postnatal exposure to GLA. As a consequence, GLA-induced abnormalities in USVs emission were likely to reflect alterations in mother–infant communicative skills. As previously said, many studies have reported unusual repertoires of USVs during infancy in mouse models of ASD, usually interpreted as communication impairments: this is the case for neuroligin-2 (Wohr et al., 2013) and neuroligin-4 (Ju et al., 2014).

Early after birth, we also observed anomalies in postnatal development in GLA1-exposed mice that is another pathologic feature frequently observed in mice models of ASD (Wohr et al., 2013). Indeed, we found that GLA1-exposed pups displayed abnormal timing of certain developmental processes. These reflexes in neonate pups are indices used to detect abnormalities in brain maturation (Fox, 1965), suggesting that the administration of GLA may have negative effects on the processes impacting on a wide range of cerebral structures. In association with impairments in social interaction, abnormal communication, and alterations in sensorimotor development constitute the core symptoms of ASD (Apa, 2000). This set of alterations are reminiscent of mice models of autism-like deficits. The effect of GLA exposure on pups’ relative brain weight also strengthened the link between autism-like deficits and pre- and postnatal exposure to GLA. Interestingly, anatomical and cellular abnormalities in the brain of ASD individuals have included reports of alterations in brain weight (Fombonne et al., 1999). Therefore, abnormalities observed in our study are in line with human studies (Courchesne et al., 2001).

In addition, pre- and postnatal exposure to GLA had a strong effect on the social skills of the offspring when adults, with their affiliative behavior showing significant alterations in three tasks classically used as behavioral phenotyping assays for mouse models of autism (Silverman et al., 2010). The laboratory mouse is a social species that engages in high levels of social interactions, sexual/parenting behaviors, and territorial/aggressive behaviors (Arakawa et al., 2008). In the present study, we showed that several aspects of the social repertoire of the mouse were affected by maternal GLA exposure, in particular sociability, i.e., inability to distinguish the social partner from the novel object in three-chambered sociability test. Male–female interactions were impaired, but no change was found in male–male interactions, suggesting that mating behavior may be specifically affected in GLA-exposed offspring. This finding tallies with reports of reduced levels of *Peg3* mRNA in the brain of GLA1 mice, as *Peg3* mutation in male mice is involved in male–female interactions (Swaney et al., 2008). This deficit was attributed to impairments in the main olfactory system rather than the accessory olfactory system. Further evidence of this can be found in animal studies, testing with nasal perfusion of zinc sulfate, which disrupts the main olfactory bulb but leaves the accessory system functionally intact (Powers and Winans, 1975). This was found to have minor effects on male–male interactions (Bean, 1982), yet completely disrupted mating behavior, regardless of whether the mice were sexually naive or experienced (Keller et al., 2006). Together, the data strongly suggest that anomalies in the main olfactory system may be involved in GLA-induced disruption of social behaviors.

Further evidence for the link between pre- and postnatal exposure to GLA and ASD-like deficits can be found in certain ASD susceptibility genes that were deregulated in exposed offspring. *Pten* is one of the many genes closely associated with autism-like symptoms, in particular with brain overgrowth and social deficits (Kwon et al., 2006; Page et al., 2009). One unexpected finding was that *Pten* mRNA levels in the brain were much lower in mice exposed to 1 mg/kg GLA, matching the increased relative brain weight in the same mice. Many reports documented the close link between *Pten* inactivation and impairments in social behaviors. Page et al., for example, reported that *Pten* haploinsufficient mice displayed abnormal affiliative behaviors associated with brain overgrowth (Page et al., 2009); while conditional *Pten* null mice displayed impaired social interactions and no preference for social novelty (Kwon et al., 2006). Even if the primary goal of the present study was not to unraveling molecular mechanisms involved in disturbing effects of pre- and postnatal exposure to GLA, our molecular data strengthened the hypothesis of a link between early life exposure to pesticides, in our case the herbicide GLA, and autism-like deficits.

Considering all our data, we need to address the question of whether our findings in mice have any relevance to the link between pesticide exposure and ASDs in humans. For a number of years now, studies have shown that an unfavorable environment during early development may lead to neurodevelopmental deficits causing a broad range of lifelong problems affecting physical and mental health (Capra et al., 2013). Epidemiological investigations have recently showed that low-level pre- and postnatal exposure to pesticides is associated with abnormal behaviors and impaired mental skills (Rauh et al., 2006; Eskenazi et al., 2007). No such effects had been reported for GLA before the present study. This is now of crucial importance as one of GLA metabolites 3-methylphosphinicopropionic acid (3-MPPA) have recently been detected in the blood of pregnant women and their fetuses (Aris and Leblanc, 2011). The situation is even more disturbing as most pregnant women are exposed to many different chemicals (Woodruff et al., 2011). Circulating levels of pesticide cocktails may be asymptomatic in pregnant women, but affect their fetuses as chemicals may interfere with neurobiological substrates underlying ASD-like symptoms, in particular at critical stages of neurodevelopment. Whenever there is a genetic susceptibility to such disorders, the adverse effects of pesticides may increase the risk of the fetus and later person developing ASD-like symptoms. Our findings clearly highlight the importance to build up our knowledge of possible harmful effects of low-level pre- and postnatal exposure to pesticides. This is a relevant and topical issue as some governmental reports have noted that early exposure (pre and postnatal) to low or very low doses of pesticides is not usually covered by the tests required for regulatory approval of pesticides, and therefore that it is impossible to estimate such adverse effects (Bonnefoy, 2012).

In conclusion, we document that GLA has pervasive, harmful effects when administered during the highly sensitive pre- and postnatal periods. At the behavioral level, these effects are strikingly reminiscent of those observed in animal models of ASDs. All data presented on the effects of GLA on behavior and brain abnormalities pave the way for supplementary analyses: whole brain microarray analysis is needed to gain a better understanding of the molecular and cellular mechanisms involved in the neurodevelopmental effects of GLA. Such an analysis will no doubt contribute to our knowledge of the constellations of genes involved in pathological processes mediated by pre- and postnatal exposure to GLA. One hypothesis is that GLA (or its metabolites) acts on target-signaling systems that establish the basic patterns of connectivity, from early neuronal migration to disruption of later postnatal events that enhance or alter neuronal connections, such as dendritic growth or synapse formation/removal. The present data also raise important questions regarding the acceptable daily intake (ADI) and whether this covers the total amount of a substance that can be ingested daily over a lifetime without adverse effects. The ADI value is determined on the basis of the no observed adverse effect level (NOAEL), i.e., the level where a substance shows no toxic effects. The lowest NOAEL for GLA has been set at 2.1 mg/kg/day on the basis of on a 2-year chronic feeding study in rats (EPA, 2011). The standard procedure for regulatory testing typically involves a series of tests to determine the value. In our study, we observed harmful effects of GLA at a dose of 1 mg/kg administered three times a week during both gestation and lactation. We can thus calculate that female mice were given 3 mg/kg a week, the equivalent of 0.4286 mg/kg/day. This is approximately five times lower than the EPA approved dose (EPA, 2011). Our data can thus provide strong evidence to argue that the current NOAEL values must be revised.

## Author Contributions

Stéphane Mortaud, Céline Montécot-Dubourg, Jacques Pichon, Olivier Richard, Arnaud Menuet, Olivier Perche, Bernard Jegou, and Anthony Laugeray designed the study. Anthony Laugeray, Ameziane Herzine, Marine Aguillon-Naury, Olivier Richard, Céline Montécot-Dubourg, and Stéphane Mortaud performed pre- and postnatal exposure to glufosinate. Marine Aguillon-Naury performed toxicologic analyses in non-pregnant, pregnant, and lactating female mice. Anthony Laugeray, Ameziane Herzine, Marine Aguillon-Naury, Olivier Richard, Céline Montécot-Dubourg, Betty Hébert, and Stéphane Mortaud performed behavioral experiments/data analyses in pups. Anthony Laugeray, Ameziane Herzine, Olivier Richard, Céline Montécot-Dubourg, and Stéphane Mortaud collected brain samples from the offspring. Ameziane Herzine, Séverine Mazaud, Laurianne Lesné, and Olivier Perche performed molecular experiments/data analyses in the offspring. Anthony Laugeray and Betty Hébert performed behavioral experiments/data analyses during offspring adulthood. Anthony Laugeray wrote the manuscript. Stéphane Mortaud, Céline Montécot-Dubourg, Olivier Perche, Olivier Richard, Arnaud Menuet, Bernard Jegou, Ameziane Herzine, Betty Hébert, Séverine Mazaud, Laurianne Lesné, Sylvain Briault, and Jacques Pichon brought significant contributions to manuscript editing. Bernard Jegou, Jacques Pichon, Arnaud Menuet, Sylvain Briault, and Stéphane Mortaud performed English editing.

## Conflict of Interest Statement

The authors declare that the research was conducted in the absence of any commercial or financial relationships that could be construed as a potential conflict of interest.

## Supplementary Material

The Supplementary Material for this article can be found online at http://www.frontiersin.org/Journal/10.3389/fnbeh.2014.00390/abstract

## Abbreviations

ASD, autistic spectrum disorders; *Chd8*, chromodomain helicase DNA-binding-8; CNS, central nervous system; DCX, doublecortin; EPA, environmental protection agency; EPM, elevated plus maze; GLA, glufosinate ammonium; GLA0.2, glufosinate ammonium 0.2 mg/kg; GLA1, glufosinate ammonium 1 mg/kg; mRNA, messenger ribonucleic acid; *Peg3*, paternally expressed gene 3; PND, post natal day; *Pten*, phosphatase and tensin homolog; qRT-PCR, quantitative real-time polymerase chain reaction; USVs, ultrasonic vocalizations.
