# Supplementary material for: Pre- and Postnatal Exposure to Low Dose Glufosinate Ammonium Induces Autism-Like Phenotypes in Mice
Source: Front Behav Neurosci. 2014 Nov 20;8:390. doi: 10.3389/fnbeh.2014.00390 (PMC4238406; doi:10.3389/fnbeh.2014.00390)
Supplement: Supplementary file 1 [file Table_1.DOCX]

**Supplementary Data description**

***
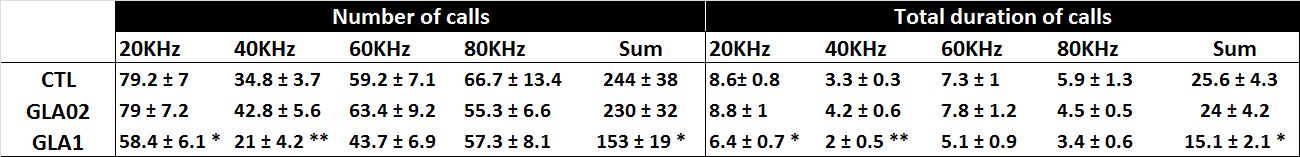
***

***Table S1:* Number and duration of ultrasonic vocalizations (USVs) emitted by pups exposed to glufosinate ammonium or saline solution.** See Materials and Methods for the detailed procedure. USVs number and duration are reduced in GLA1 (1 mg/kg) exposed offspring, in particular at 20 and 40 KHz. Values are mean +/- sem. * p<0.05 and ** p<0.01.

***Supplementary movie 1:*** Video to be uploaded online. Mice are detected by the videotracking software system (nose-tail tracking) and time the animal head spent inside the virtual “tube zone” was measured during odor presentations. See “Materials and methods” for a detailed description of the test.
